# Effects of COVID-19 Lockdown on the Emotional and Behavioral Profiles of Preschool Italian Children with and without Familial Risk for Neurodevelopmental Disorders

**DOI:** 10.3390/brainsci11040477

**Published:** 2021-04-09

**Authors:** Chiara Cantiani, Chiara Dondena, Elena Capelli, Elena M. Riboldi, Massimo Molteni, Valentina Riva

**Affiliations:** Child Psychopathology Unit, Scientific Institute, IRCCS Eugenio Medea, Bosisio Parini, 23842 Lecco, Italy; chiara.dondena@lanostrafamiglia.it (C.D.); elena.capelli@lanostrafamiglia.it (E.C.); elenamaria.riboldi@lanostrafamiglia.it (E.M.R.); massimo.molteni@lanostrafamiglia.it (M.M.); valentina.riva@lanostrafamiglia.it (V.R.)

**Keywords:** COVID-19, children, neurodevelopmental disorders, familial risk, emotional and behavioral development

## Abstract

The effects of COVID-19 containment measures on the emotional and behavioral development of preschoolers are not clear. We investigated them within an ongoing longitudinal project including typically developing children (TD) and children at high familial risk for neurodevelopmental disorders (HR-NDD) who were potentially more vulnerable. The study included ninety children aged 2–6 years (TD = 48; HR-NDD = 42). Before the emergency phase (T0), all children received a clinical assessment, including the parent questionnaire Child Behavior Checklist for Ages 1.5–5 (CBCL 1.5–5). The same questionnaire was filled out again during the emergency (T1), together with an ad-hoc questionnaire investigating environmental factors characterizing the specific period. Changes in the CBCL profiles between T0 and T1 were evaluated. Overall, irrespective of familial risk, the average T-scores on specific CBCL scales at T1 were higher than at T0. Associations emerged between delta scores reflecting worsening scores on specific CBCL scales and clinical and environmental factors. Our results confirmed the negative impact of the lockdown on preschool children’s emotional/behavioral profiles, and highlight the need for strategic approaches in the age range of 2–6 years, especially for more susceptible children owing to environmental factors and pre-existing emotional problems.

## 1. Introduction

To mitigate the spread of COVID-19, Italy, like many other countries, introduced unprecedented measures, including abrupt cessation of childcare services, lockdown, and social distancing. In particular, a national lockdown was imposed by the Italian government on 11 March 2020 (“Phase 1”), suspending all non-essential activities and allowing people to leave their houses to fulfill primary needs only. These restrictions were then partially lifted from 4 May (“Phase 2”). Clearly, these measures can negatively impact the psychological well-being of children for several reasons. Owing to childcare and school closure, children had to stay at home for extended periods and had minimal interaction with their peers [1]. This is problematic, given that during childhood peer contact is extremely important for well-being [2]. A recent systematic review found an association between loneliness and mental health problems-including anxiety and depression-in children and adolescents, suggesting that the current social distancing measures could cause an increase in such mental health problems [3]. In addition, containment measures have led to a lack of daily routine and structure and children may struggle to cope with sudden changes. Importantly, keeping a routine produces a sense of discipline and safety in children, which is important for their psychological development [4]. Evidence suggests that children become physically less active, have much-prolonged screen time and irregular sleep patterns even during weekends and summer holidays, when they have a less structured life schedule [5,6]. Such negative effects are likely to be much worse during the lockdown, given its length duration and since children are confined to their homes without outdoor activities and interaction with same-aged friends [6]. The sudden re-organization of familial everyday life causes enormous psychological distress not only in children but also in the whole family. All family members have to cope with the stress of lockdown and social distancing. According to a recent review, the main pandemic stressors during lockdown are fear of infection, dissatisfaction and boredom, lack of adequate information or clear guidelines from public authorities, lack of personal space at home, and family’s financial loss [7]. In addition, during lockdown family connections are disrupted, external support is lacking and families put much energy in balancing childcare, education and support to teaching on one hand, and to work responsibilities on the other hand. It is clear that all these factors together can negatively affect children, with an enduring impact on their mental health [7].

One study has investigated psychosocial responses in children isolated or quarantined during previous pandemic diseases and found that children were more likely to develop acute stress disorder, adjustment disorder, and grief, with 30% of them meeting the clinical criteria for post-traumatic stress disorder [8]. The first investigations conducted during the COVID-19 outbreak revealed a wide variety of mental health issues in children and adolescents, including anxiety and depression [9,10,11,12], sleeping disorders [1,10], irritability and difficulty in concentrating [1,13,14], clinginess and excessive dependence on parents [1,13] and worries and fear that family members could contract the infection [1,13,15]. A recent meta-analysis by Panda et al. [16] revealed that 34.5%, 41.7%, 42.3% and 30.8% of the children were suffering, respectively, from anxiety, depression, irritability and inattention. One study specifically compared children from the general population—both younger and older than 6 years of age—and suggested that clinging, inattention and irritability were the most severe psychological conditions displayed by children in all age groups, whereas children in the younger age group (3–6 years) were more likely than older children to manifest symptoms such as clinginess and fear that family members may contract the infection [1].

In the current situation, some factors do increase the vulnerability of children to psychological problems. These include direct contact with illness (i.e., being separated from caregivers who are infected or suspected of being infected, and having caregivers/relatives infected with the disease or who died because of the disease) [10,17] and level of parental stress [13,18], as well as depressive symptoms in parents [10]. Other effects associated with children’s psychological problems are higher parental education level, current parental occupation (no remote work), and living in an urban area [9,10]. To the best of our knowledge, the largest epidemiological study was carried out in China on 12,163 children aged 2–5 years and 17,029 children aged 6–12 years [19], showing that psychosocial problems were higher in children with special educational needs, and/or acute or chronic disease, mothers with mental illness, single-parent families, and low-income families.

There are several indicators that children with pre-existing vulnerabilities and disadvantages are at highest risk and more likely to be disproportionately affected [20,21,22,23,24]. Social distancing measures are difficult to understand for children, especially for those experiencing developmental delays [21]. Social distancing and lack of outdoor activities deteriorate the development of children with impairment in social skills [21]. Children with learning difficulties and neurodevelopmental disorders (NDD) might be even more affected by changes and disruptions to routines than typically developing (TD) peers [23]. For children with mental health issues, school routines are important coping mechanisms and anchors in life: school closure could cause a relapse of their symptoms [22]. Children with Autism Spectrum Disorder (ASD) are also at risk, since lack of routines can make them feel more anxious, frustrated, irritable and restless [21,22,24]. In addition, school closures and lockdown deprived many children with mental health needs and NDD of access to resources that they usually get through schools [22] and face-to-face therapies [24,25]. Even if most educational and support systems have shifted to online telehealth programs [26,27], these platforms may not be compatible with assistive technology [21] and may be challenging for some young people [22]. Families have been asked to take care of online special education and therapies, which have further increased parents’ load and psychological distress, with negative effects on children [20,21]. All these factors together put children at greater risk of relapse or worsening of mental health issues [28,29,30,31]. According to a cross-sectional U.K. study, during the COVID-19 emergency children with NDD (aged 4–15 years) had worse emotional symptoms compared to a similar pre-COVID-19 mental health cohort and a higher prevalence of emotional symptoms and conduct problems compared to neurotypical controls [32]. A similar cross-sectional study was conducted in Italy [33]. In this study, the authors compared a sample of 82 children with NDD (age 3–17 years) to age-matched TD children. Although the two groups of children differed at baseline in terms of parental stress and externalizing behaviors (with children with NDD showing higher measures), both groups increased in the two measures during the emergency phase compared to before, independent of the children’s diagnostic status. Conti et al. [27] conducted an observational longitudinal study to investigate lockdown-related emotional and behavioral changes in the pediatric neuropsychiatric population, including NDD. Increased anxiety and somatic problems were reported in younger children (1.5–5 years, *n* = 61), whereas obsessive-compulsive, post-traumatic and thought problems increased in older children (6–18 years, *n* = 80).

To date, evidence of the effects of COVID-19 lockdown on the emotional and behavioral profiles of young children (below 6 years of age) is limited and mainly based on cross-sectional investigations (information on pre-emergency behaviors was collected retrospectively during the emergency). There is no evidence yet of the potential vulnerability of younger children who are at higher familial/biological risk for NDD. Given the high heritability of NDD [34,35], there is a greater-than-expected prevalence of such disorders in infants and toddlers siblings of children with a clinical diagnosis [36,37], which is defined as at-high familial and biological risk. Here, we investigate these effects within a currently ongoing longitudinal project including TD children and children at high familial risk for NDD (HR-NDD), including children having a first-degree relative with ASD, Developmental Language Disorder (DLD) or Learning Disabilities, who are potentially more vulnerable. For both groups of children (TD and HR-NDD) data were collected within a longitudinal design, providing measures at two different times: before and during the pandemic. Effects of the COVID-19 lockdown on children’s emotional behavioral profiles were investigated through the Child Behavior Checklist for Ages 1.5–5 (CBCL) [38], based on (1) familial/biological risk for NDD and (2) clinical, socio-demographic and environmental factors. Starting from previous reports of negative effects of the COVID-19 lockdown on the general child and adolescent population and of the additional challenges for children with NDD, we expected worsening of emotional and behavioral profiles in all children and a more marked worsening in children at risk for NDD. Based on the previous literature, we expect worsening in specific CBCL scales, reflecting anxiety and depression [9,10,11,12,16,27], sleeping disorders [1,10], difficulty in concentrating [1,13,14,16], irritability and externalizing behaviors [1,13,14,16,33], and somatic complaints [27]. In addition, we hypothesized that some clinical, socio-demographic and environmental factors might act as protective or risk factors. Although it is hard to have clear theoretically-driven predictions based on the limited existing research, we hypothesize the following factors to increase the vulnerability of children to psychological problems and thus to act as risk factors: (a) worse clinical symptoms, as suggested by the literature on children with a full-blown diagnosis of NDD [27,32,33]; (b) presence of psychological and behavioral problems before the emergency, as expressed by internalizing and externalizing behaviors; (c) low parental education and income [9,10,19], and (d) direct contact with illness [10,17]. Conversely, we hypothesize the following variables to act as protective factors: (a) presence of adequate space at home, particularly relevant since children were not allowed to exit from their houses [39]; (b) adequate explanations of the situation given to children, enabling them to make sense and accept the containment measures; and (c) contact with kindergarten, allowing children to maintain minimal interaction with their peers [2].

## 2. Materials and Methods

The study sample was recruited within an ongoing longitudinal project aiming at identifying early risk markers for NDD. Specifically, the longitudinal study included children from the general population [40,41] and children with a first-degree relative (i.e., a sibling) with a clinical diagnosis of ASD, DLD or Learning Disabilities [42,43].

Children were recruited when they were younger than 12 months of age in the area of Lecco, Como and Monza-Brianza (Northern Italy). Individual and socio-demographic information such as gestational age at birth, socioeconomic status (SES), and parental education level was collected during the first visit (Table 1). SES was scored according to Hollingshead 9-point scale, whereby a score ranging 10–90 was assigned to each parental job and the higher of two scores was used when both parents were employed [44]. Parental education level was scored on a 9-point ordinal scale, which had been created ad-hoc and was based on the Italian school system. In addition, follow-up visits including standardized assessment and parent-report measures were scheduled at 18, 24, 36 and 48 months of age.

Parents of 188 children aged 2–6 years included in the main longitudinal project were asked to take part in the present study. They had previously filled the CBCL 1.5–5 [38] at least once within the longitudinal study (data collection hereinafter referred to as Time “T0”), and were asked to fill it out again for the purpose of the present study (hereinafter referred to as Time “T1”).

Ninety families participated in the present study (T1 data collection), completing the full questionnaire within the requested time frame (see Section 2.3, Procedure). Although participating families (i.e., families who completed the full questionnaire at T1) and non-participating families (i.e., families who did not complete the full questionnaire at T1) did not differ in gestational age at birth, parental education level and sex (*p_s_* > 0.09), differences were found for SES (higher scores, corresponding to higher status, in participating families: *M* = 64.65, *SD* = 17.54, than in non-participating families: *M* = 56.18, *SD* = 19.70; *t*(176) = −3.01, *p* = 0.003, *d* = −0.453) and family history for NDD (higher prevalence of TD in participating families than in non-participating families, χ^2^(1, *n* = 188) = 11.944, *p* = 0.001).

The final sample consisted of 48 TD children (27 males) and 42 HR-NDD children (24 males) for the above-mentioned disorders (specifically, 28 children at risk for ASD; 14 children at risk for DLD and/or Learning Disabilities). The two groups did not differ for sex (χ^2^(1, *n* = 90) = 0.007, *p* = 0.932; see Table 1 for a complete description of the sample in terms of individual and socio-demographic information).

### 2.1. Measures

#### 2.1.1. Child Behavior Checklist for Ages 1.5–5

The CBCL 1.5–5 is a 99-item parent-report measure designed to record emotional and behavioral problems in toddlers [38]; Italian adaptation by Frigerio et al. [45]. Each item describes a specific behavior and is scored on a 3-point Likert scale (0 = not true; 1 = sometimes true; 2 = very true). The scoring produces seven Syndrome Scales (Emotionally Reactive, 9 items; Anxious/Depressed, 8 items; Somatic Complaints, 11 items; Withdrawn, 8 items; Sleep Problems, 7 items; Attention Problems, 5 items; Aggressive Behavior, 19 items), clustered in a summary profile made by Composite Scales (including Internalizing, Externalizing, and Total Problems scores), and five scales related to DSM-IV disorders (Affective Problems, Anxiety Problems, Pervasive Developmental Problems, Attention Deficit/Hyperactivity Problems and Oppositional Defiant Problems). This measure showing strong psychometric properties across cultures was translated into, and validated in Italian [46]. During the overall longitudinal project (T0), parents were asked to answer the questionnaire based on the previous two months, whereas at T1 parents were asked to refer to the last month. T-scores (mean = 50; *SD* = 10) were used in the analysis. Higher scores indicate greater problems.

#### 2.1.2. Environmental Questionnaire

A questionnaire to investigate socio-demographic and environmental factors characterizing the specific period of assessment was drafted. It included multiple-choice questions about (a) the environment in which children spent the lockdown, i.e., size of their house in terms of number of rooms; direct access to exteriors, i.e., presence of balcony or garden; people they were in direct contact with during the lockdown, i.e., grandparents, babysitter; (b) type of explanation, if any, given to children regarding the pandemic; (c) presence of any contact with kindergarten during lockdown, via video-calls or assigned activities; (d) parental occupation during lockdown, i.e., whether they continued to go to workplace, they worked from home, their activity was suspended, or they lost their job (this variable was inserted to check for changes in parental occupation leading to changes in SES); (e) any family members or friends infected with COVID-19.

#### 2.1.3. Language and Social Communication Assessment

Language and social communication variables for each participant were previously collected during the main longitudinal project (T0). At 18, 24, 36 months of age, percentile scores for expressive vocabulary were collected via parent-report surveys, in which parents were asked to identify from a list of words those that their children used spontaneously (Language Development Survey, LDS [47], *n* = 57; or ‘Primo Vocabolario del Bambino, PVB, Parole e Frasi’ [48], *n* = 33). Only the most recent score available was considered for this study.

At 18 months of age, social communication skills were measured through the Modified Checklist for Autism in Toddlers (M-CHAT) questionnaire [49], which requires parents to report on the presence of specific child behaviors in a 23-item checklist in order to detect ASD-related traits. Three or more failed items are indicative of the presence of ASD symptoms.

### 2.2. Procedure

Parents were asked to complete the CBCL 1.5–5 and the ad-hoc environmental questionnaire between April 17th and May 4th, 2020 (during “Phase 1” of the Italian emergency). These scores were entered in the analyses as T1 measurements (mean = 43.71 months, *SD* = 13.21, min = 25, max = 71). For each child with complete data at T1, the most recent CBCL 1.5–5 collected prior to the emergency (i.e., up to February 2020) was considered, and scores were entered into the analyses as T0 measurements (mean = 31.90 months, *SD* = 11.58, min = 18, max = 63). The time range between T0 and T1 was 11 months on average (min = 2, max = 43).

### 2.3. Statistical Analysis

To compare the two groups pre-pandemic, independent samples *t*-tests were performed to assess differences in clinical and CBCL scores at T0. Welch-*t* test *t*-values and *p*-values were reported when the equality of variance assumption was violated. Pearson-χ^2^ group difference analyses were then performed to assess differences in the variables from the ad-hoc questionnaire, to characterize the two groups’ environment during Phase 1 of the emergency. To evaluate changes in the CBCL scales, 2 × 2 × 7, 2 × 2 × 3 and 2 × 2 × 5 repeated-measure ANOVAs were performed, including the between-subject factor Group (2 levels: TD vs. HR-NDD) and the within-subjects factors Time (2 levels: T0 vs. T1) and Scale (7 levels for Syndrome Scales; 3 levels for Composite Scales; 5 levels for DSM-oriented Scales). Greenhouse-Geisser-corrected *p*-values were reported when appropriate. Significant interactions were further explored by means of paired *t*-tests by comparing the two time points (T0 vs. T1) for each scale. For each set of scales, the significance alpha threshold was adjusted to account for multiple testing (Syndrome Scales: 0.05/7 = 0.007; Composite Scales: 0.05/3 = 0.017; DSM-oriented Scales: 0.05/5 = 0.01). For those scales that had statistically different scores between the two time points in the expected direction (i.e., worsening at T1 with respect to T0), a difference score was obtained (T-scores at T1 minus T-scores at T0). This difference score was used to investigate Pearson or Spearman’s correlations with (a) clinical variables relative to linguistic and social communication skills, previously collected during the main longitudinal study, (b) pre-emergency psychological and behavioral problems, quantified as the Internalizing and Externalizing scores obtained for the CBCL composite scales at T0; (c) environmental scores obtained from the main longitudinal study, specifically parental education and SES; (d) environmental information collected at T1 through the ad-hoc environmental questionnaire, including (i) direct contact with illness, (ii) presence of adequate space at home, (iii) explanations of the situation given to children, and (iv) contact with kindergarten. For each set of correlations, the significance alpha threshold was adjusted to account for multiple testing.

## 3. Results

### 3.1. Sample Characteristics at T0

The two groups were compared on the clinical scores collected within the main longitudinal project (most recent scores available). As expected, children in the TD group had a larger expressive vocabulary percentile score compared to children in the HR-NDD group (TD, *M* = 57.92, *SD* = 24.43; HR-NDD, *M* = 33.33, *SD* = 27.29; *t*(88) = 4.510, *p* < 0.001, *d* = 0.953). The number of failed items in the M-CHAT social communication score was not significantly different between groups, although slightly higher in the HR-NDD group (*M* = 1.13, *SD* = 1.58) compared to the TD group (*M* = 0.70, *SD* = 1.13) (*t*(88) = 1.468, *p* = 0.146, *d* = 0.320). Finally, four HR-NDD children had already been diagnosed with ASD; three children had been diagnosed with DLD, two of them belonging to the HR-NDD group and one to the TD group.

Regarding emotional and behavioral profiles before the emergency, the CBCL scores of the two groups at T0 were compared via independent samples *t*-tests and no significant differences emerged on any of the scales (see Table S1 in the Supplementary Materials), suggesting that the two groups had similar baseline measures.

### 3.2. Sample Characteristics at T1 (COVID-19 Emergency Phase)

Table 2 shows the sample characteristics and group differences for the environmental measures collected through the ad-hoc questionnaire. Chi-squared analyses comparing the two groups showed no significant difference in the variables regarding (a) the environment in which children spent the lockdown, (b) presence/absence of any contact with kindergarten during lockdown, (c) father’s occupation during lockdown, (d) family members or friends infected by COVID-19 (see Table 2, *p* > 0.05). Conversely, a significant difference was found in the quantity of explanations given to the children about the pandemic (χ^2^(2, *n* = 90) = 7.220, *p* = 0.027), showing that children in the HR-NDD group were more likely to receive no explanation at all (31% of HR-NDD children vs. 10.4% of TD children). A significant difference was also found in the variable regarding maternal occupation during lockdown (χ^2^(5, *n* = 90) = 12.090, *p* = 0.034), showing that mothers of TD children were more likely to continue to go to their workplace during lockdown compared to mothers in the HR-NDD group (TD = 29.2% vs. HR-NDD = 11.9%). Mothers in the HR-NDD group were more likely to be unemployed/housewives since before the emergency phase (TD = 16.7% vs. HR-NDD = 31%).

### 3.3. Changes in the Emotional and Behavioral Profiles during the Emergency Phase vs. Pre-Emergency

Repeated-measures ANOVAs revealed a significant Time *x* Scale interaction for the Syndrome Scales (F(4, 376) = 6.243, *p* < 0.001, *ŋ*^2^ = 0.066), for the Composite Scales (F(1, 108) = 6.905, *p* = 0.006, *ŋ*^2^= 0.073) and for the DSM-oriented Scales (F(3, 308) = 7.730, *p* < 0.001, *ŋ*^2^ = 0.081). No significant main effect of group and interactions involving group emerged (*p_s_* > 0.05), suggesting that changes in emotional and behavioral profiles during the emergency phase vs. pre-emergency were not modulated by biological/familial risk.

**Figure 1:**
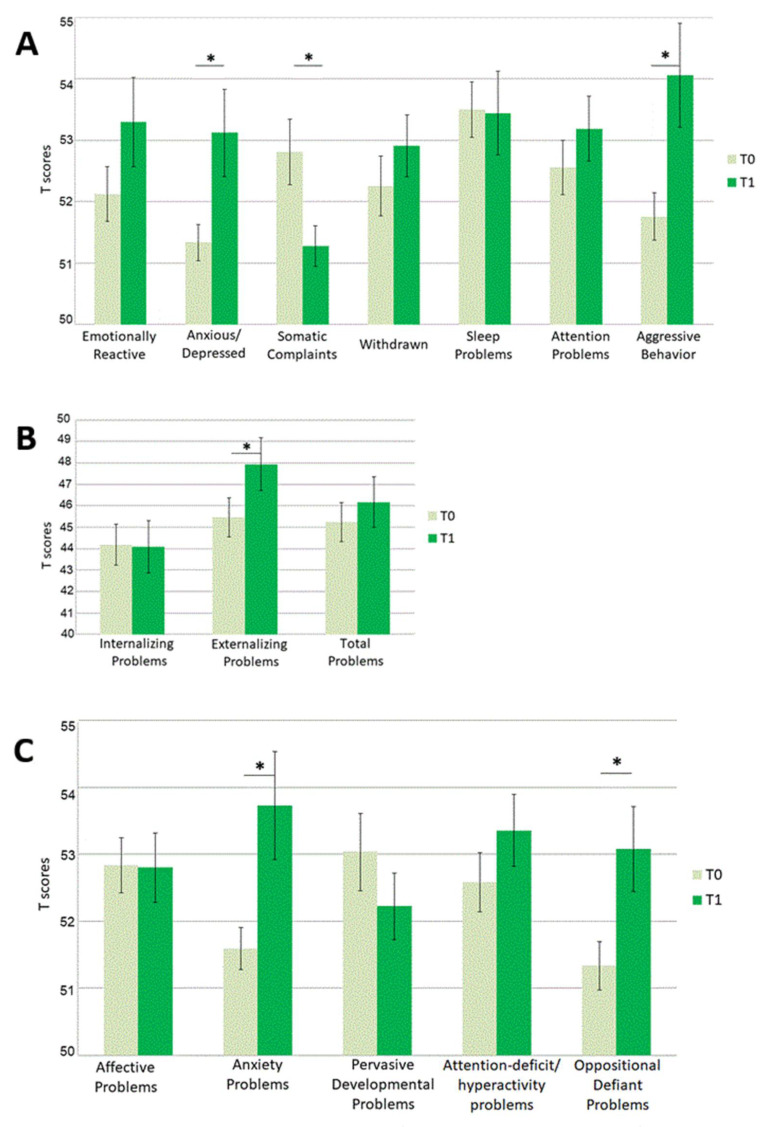
Descriptive graphical representation of mean T-scores (and Standard Errors) for each CBCL 1.5–5 scale. T-scores at T0 (grey bars) are plotted against T-scores at T1(green bars) for (**A**) Syndrome scales, (**B**) Composite scales, and (**C**) DSM-oriented scales. Asterisks (*) indicate significant differences between T-scores at T0 and at T1.

Given the absence of any effect of Group, the sample was considered as a whole for the following analyses. Paired *t*-tests contrasting T-scores at T0 vs. T-scores at T1 on each scale revealed significant differences for the following scales: Anxious/Depressed (*t*(89) = −2.884, *p* = 0.005, *d* = −0.304), Somatic Complaints (*t*(89) = 3.382, *p* = 0.001, *d* = 0.357) and Aggressive Behavior (*t*(89) = −2.931, *p* = 0.004, *d* = −0.309) among Syndrome scales; Externalizing Problems (*t*(89) = −2.476, *p* = 0.015, *d* = −0.261) among composite scales; Anxiety Problems (*t*(89) = −3.114, *p* = 0.002, *d* = −0.328) and Oppositional Defiant Problems (*t*(89) = −3.002, *p* = 0.003, *d* = −0.316) among DSM-oriented scales. All above mentioned comparisons survived correction for multiple comparisons. For each of these scales, except for the Somatic Complaints scale, T scores were higher at T1 (see Figure 1, Table S2).

### 3.4. Associations between Changes in Emotional and Behavioral Profiles and Clinical/Environmental Variables

To investigate associations between worsening emotional and behavioral characteristics and clinical or environmental variables, a difference score (T1–T0) was calculated for the CBCL scales reported above (Anxious/Depressed and Aggressive Behavior among Syndrome scales; Externalizing Problems among Composite Scale; Anxiety Problems and Oppositional Defiant Problems among DSM-oriented Scales). The difference found in the Somatic Complaints scale was not included in the correlation analysis, since we were specifically interested in checking for the factors associated with worsening CBCL scales. Since we included five CBCL scales in each set of correlations, the significance alpha threshold was adjusted to account for multiple testing (0.05/5 = 0.01).

When considering the sample characteristics before the emergency (T0), no significant correlation was found with clinical scores, i.e., expressive vocabulary and socio-communication scores (*p* > 0.01); conversely, significant correlations were found with the CBCL Composite scales at T0 (Internalizing Problems scale), showing that the higher this score was at T0, the higher the difference scores were for the five scales considered (Table 3). Specifically, the correlations surviving conservative correction for multiple comparisons were the associations between Internalizing Problems at T0 and Aggressive Behavior, Anxiety Problems, and Oppositional Defiant Problems at T1.

Although no significant Spearman’s correlations were found with the environmental variables collected within the main longitudinal project (namely parental education and SES, see Table 4), a few associations emerged for the specific environmental variables collected through the ad-hoc questionnaire during the emergency phase (T1) (see Table 4). The resulting associations could be summarized as follows: the bigger the children’s house was in terms of number of rooms, the lower the increase in anxiety scores at T1; the more they had access to exteriors (e.g., presence of balcony or garden), the lower the increase in externalizing and oppositional problems at T1; the more explanations they received, the greater the increase in anxiety/depression issues at T1; finally, if they had any contact with kindergartens, they were more likely to have less externalizing problems at T1. This latter association was the only one surviving conservative correction for multiple comparisons (0.05/5 = 0.01).

## 4. Discussion

The present study aimed at exploring the effects of COVID-19 lockdown on the emotional behavioral profiles of Italian preschoolers. Specifically, we were interested in the role played by familial/biological risk for NDD and by clinical, socio-demographic and environmental factors. To date, few studies have specifically focused on children younger than age 6 years [1,19,27], and no study has focused on HR-NDD children. Here we showed that during the COVID-19 emergency, Italian preschoolers aged 2 to 6 years exhibited increased anxiety, depression, and externalizing problems, including aggressive behaviors and oppositional defiant problems. Contrary to our expectations, the impact of the COVID-19 lockdown affected all children equally, without being prevalent among HR-NDD children to a greater extent. Among clinical, socio-demographic and environmental factors who might affect the degree of emotional and behavioral problems during the emergency, we found that pre-existing vulnerabilities towards internalizing problems seemed to act as a risk factor, whereas having any contact with kindergarten seemed to act as a protective factor.

Our hypothesis of a greater vulnerability of children at familial risk for NDD was driven by existing studies reporting or expecting a higher negative impact on children with a full-blown diagnosis of NDD [27,33,50] compared to TD children. Contrary to our expectations, the negative effects of COVID-19 lockdown on children’s emotional behavioral profiles were not more pronounced for HR-NDD children compared to TD children. The two groups did not differ in terms of age and baseline measures relative to emotional behavioral profiles. They differed for clinical measures (i.e., language measures were lower in the HR-NDD group compared to the TD group) and some environmental variables pre-emergency (i.e., SES and maternal education were lower in the HR-NDD group). They also differed for two environmental variables during emergency: (1) maternal working situation during lockdown, with mothers in the HR-NDD group more likely to be unemployed/housewives since before the emergency and mothers of TD children more likely to continue to go to workplace during lockdown; and (2) explanations of the situation given to children, with children in the HR-NDD group more likely to receive no explanation. Despite these differences, which were most penalizing the HR-NDD group, the two groups showed no differences in terms of COVID-19 impact on their emotional and behavioral profiles: effects were not more pronounced on HR-NDD children compared to TD children. This result was contrary to our expectations based on previous studies conducted on children with full-blown diagnosis of NDD [27,32,33,50]. In order to interpret this apparently contrasting finding, it is important to keep in mind that only a restricted proportion of HR-NDD children in our sample had already received a clinical diagnosis (i.e., *n* = 6 in total, 4 children diagnosed with ASD and 2 children with DLD, respectively, corresponding to 14% of the total HR-NDD group) and were receiving a therapy that was suddenly interrupted and/or switched online. It could be hypothesized that discontinuation of face-to-face therapies may be one of the main reasons for the greater vulnerability of children with NDD to emotional and behavioral difficulties during the COVID-19 emergency reported by previous studies [27,32,33,50]. Bentenuto et al. [33] have shown that the increase in externalizing behavior in children with NDD was indeed related to a decrease in provided therapies.

We then investigated which aspects of the children’s emotional and behavioral profiles were more affected. With regards to the seven Syndrome Scales, scores on the Anxious/Depressed and Aggressive Behavior scales worsened vs. baseline. Surprisingly, scores on the Somatic Complaints scale improved vs. baseline. When considering a summary profile consisting of Composite Scales, only externalizing behaviors were more affected. From a clinical DSM-IV-oriented point of view, the Anxiety Problems and Oppositional Defiant Problems scales showed worsening scores. Taken together, these results were in line with previous investigations reporting anxiety and depression [9,10,11,12,27] and irritability and/or externalizing behaviors [1,13,14,33] as the main mental health issues affecting children and adolescents during the COVID-19 outbreak [16]. Unlike previous literature, we did not find specific problems related to sleep patterns [1,10] and attention [1,13,14]. With respect to previous studies focusing on children younger than 6 years of age [1], we did not find symptoms ascribable to clinginess and excessive dependence on parents. This might be because the CBCL 1.5–5 was not the most appropriate instrument to investigate this specific aspect. It is interesting to compare our results with a study by Conti et al. [27] employing the same measure (CBCL 1.5–5) on an Italian neuropsychiatric pediatric population. Consistent with our findings, the authors found a significant increase in the DSM-Oriented Anxiety Scale scores. Contrary to our findings, they found an increase in somatic problems. The somatic complaints point to physical symptoms that are not better explained by a medical condition and can thus be defined as an expression of a psychological difficulty. As reported by parents, the prevalence of somatization in preschool children ranges between 20 and 30% [51,52,53]. It might be hypothesized that school-related stress is one of the main causes of such symptoms in children attending kindergarten, as already reported in older children and adolescents [54]. It is hard to interpret the opposite findings related to somatic complaints in Conti et al. [27]. It should be noted that the population included in the two studies–although identical for age–was not identical for type of disorders (TD children or HR-NDD children–but without full-blown diagnosis in our study vs. a neuropsychiatric population in Conti et al. [27]).

To identify the factors responsible for a greater vulnerability to psychological problems in the current situation, we computed correlations between a difference score reflecting worsening scores on scales with statistically different scores between the two time points and (a) clinical variables relative to linguistic and social communication skills, (b) pre-emergency psychological and behavioral problems, (c) pre-emergency environmental scores (i.e., socio-demographic information), (d) emergency environmental information collected at T1. Consistently with the finding of no increased vulnerability in the HR-NDD group, we did not find any associations between worsening CBCL scale scores and clinical variables including expressive vocabulary and social communication scores. Conversely, significant associations were found with pre-emergency CBCL Internalizing Problems scale scores: children who experienced more psychological problems during the emergency (specifically more aggressive behaviors, anxiety problems, and oppositional defiant problems) were children showing more internalizing problems before the emergency. This supports the hypothesis that children with pre-existing vulnerabilities and disadvantages are more likely to be disproportionately affected.

Regarding environmental variables, we only found that some emergency-related variables were associated with emotional and behavioral symptoms during the emergency. Specifically, in our study having any contact with kindergarten during lockdown (e.g., via video-calls or assigned activities) seemed to act as a protective factor against externalizing problems. These findings were consistent with recent studies conducted on ASD children and reporting that children who did not receive school support since the start of the COVID-19 lockdown expressed more intense behavioral problems than those who did [28] and confirming that school support and contact with friends and family during the lockdown period protected against worsening social skills [55]. Contrary to previous reports [10,17], we did not find significant correlations including direct contact with illness (i.e., having relatives or friends infected by COVID-19). The other associations found did not survive correction for multiple comparisons, and thus should be taken cautiously. However, given the exploratory nature of the work, we are reporting and interpreting them. First, we found a trend indicating that the environment in which children spent the lockdown affected their psychological problems: specifically, the higher number of rooms and having more physical space for themselves seemed to act as protective factor against anxiety, whereas direct access to exteriors seemed to act as protective factor against externalizing and oppositional problems. These findings are not surprising, since previous studies have already demonstrated associations between housing conditions and psychological and people’s well-being and mental health in general [56] and specifically with the behavioral symptomatology during COVID-19 lockdown, both in adults [57] and in children [39]. Second, we found a trend indicating that explanations on the pandemic given to children were surprisingly positively correlated with an increase in anxiety/depression problems: children who received more information by parents were those suffering more. The importance of supporting parents with strategies enabling them to communicate correctly with their children about the pandemic should be taken into serious consideration when defining guidelines for reducing the impact of lockdown in young children. Clearly, communicating with children about COVID-19 should be a priority, yet quantity and quality of information should take into account the child’s age and level of understanding [58,59]. Parents need to help their children interpret the large amounts of potentially confusing information they receive and to cope with unclear messages [60].

Some limitations of the present study should be mentioned. First, the sample size compared to recent multicentric epidemiologic studies was limited [19]. We cannot exclude that some small effects that would be significant in a larger sample were missed here due to the limited sample size. However, it should be noted that we did not intend to undertake an epidemiological study, but rather to describe the effects of the COVID-19 lockdown in a small but well characterized longitudinal sample. Second, we should acknowledge that our selection of socio-demographic and environmental variables potentially affecting psychological and behavioral problems during lockdown was not exhaustive. For example, the economic impact of the pandemic on the participating families-in terms of changes of family income-was not assessed, although it has been reported to have an effect on children’s mental health [9,10,19]. Third, we used a self-reported parental measure (CBCL 1.5–5). The parents’ own level of psychological distress in facing the pandemic–information that we did not directly collect-may have interfered with their responses to their children’s functioning. Finally, participating and non-participating families differed in terms of SES and prevalence of HR-NDD. Parents that were able and willing to participate had a higher SES. In addition, there was a higher proportion of TD families (63%) vs. HR-NDD families (37%) among participating families. This possible selection bias should be kept in mind when interpreting results.

Besides the limitations, however, the strengths of our study should be acknowledged. The main strength of the study is that data were collected within an ongoing longitudinal project providing measures at two different times (T0 and T1), which offered a unique opportunity to study the effects of the pandemic situation on the children’s emotional and behavioral development. Specifically, (1) baseline measures of participating children before the start of the COVID-19 emergency phase and (2) a full range of clinical measures were available. Such measures were available both for children from the general population and for HR-NDD children, enabling a direct comparison between groups.

## 5. Conclusions

To the best of our knowledge, this is the first study offering empirical results on the effects of COVID-19 lockdown on preschoolers with and without familial risk for NDD. Our results demonstrated the negative impact of the COVID-19 lockdown on the emotional and behavioral profiles of Italian preschoolers, irrespective of the risk for NDD, but with a worse effect on children with previous emotional and behavioral vulnerabilities. These results provide initial information for interventions and strategic approaches in the age range of 2–6 years of age. Since the beginning of the pandemic, clinical activities have been remotely reorganized to reduce impact on the National Health System. A working model in telemedicine has been developed [61] and could be especially helpful for children with higher susceptibility to sociodemographic and environmental factors and previous emotional and behavioral problems.

## Figures and Tables

**Table 1:** Descriptive statistics: Mean (Standard Deviation) and group comparisons on individual and socio-demographic variables.

	TD (*n* = 48)	HR-NDD (*n* = 42)	*t* (df)	*p*	Cohen’s *d*
Gestational age (weeks)	39.09 (1.29)	38.98 (1.61)	0.371 (78)	0.712	0.083
Socioeconomic status ^a^	68.37 (15.49)	60.26 (18.95)	2.172 (83)	**0.033**	0.473
Maternal education level ^b^	61.28 (13.61)	54.38 (16.06)	2.170 (85)	**0.033**	0.467
Paternal education level ^b^	49.36 (16.47)	46.75 (15.26)	0.762 (85)	0.448	0.164

^a^ 9-point scale, whereby a score ranging 10–90 was assigned to each parental job and the higher of two scores was used when both parents were employed [44]. ^b^ 9-point ordinal scale, created ad-hoc and based on the Italian school system. In bold the significant differences between groups.

**Table 2:** Sample characteristics and group differences related to the ad-hoc environmental questionnaire.

	Total	TD	HR-NDD	χ^2^ (df)	*p*
**Access to exteriors (%)**				1.44 (2)	0.487
No private access to exteriors	8 (8.9)	3 (6.3)	5 (11.9)		
Balcony/Terrace	34 (37.8)	17 (35.4)	17 (40.5)		
Garden	48 (53.3)	28 (58.3)	20 (47.6)		
**Size of the house-N of rooms (%)**				4.31 (4)	0.365
2	3 (3.3)	0 (0.0)	3 (7.1)		
3	18 (20.0)	10 (20.8)	8 (19.0)		
4	34 (37.8)	17 (35.4)	17 (40.5)		
5	19 (21.1)	11 (22.9)	8 (19.0)		
More than 5	16 (17.8)	10 (20.8)	6 (14.3)		
**Contact with kindergarten (%)**				3.68 (1)	0.055
No	16 (18.4)	5 (11.9)	11 (26.8)		
Yes	71 (81.6)	41 (89.1)	30 (73.2)		
**Explanation(s) given to the child (%)**				7.22 (2)	**0.027**
None	18 (20.0)	5 (10.4)	13 (31.0)		
One type	49 (54.4)	27 (56.3)	22 (52.4)		
Two or more types	23 (25.6)	16 (33.3)	7 (16.7)		
**Mother’s working status during lockdown (%)**				12.1 (5)	**0.034**
Homemaker/not employed since before lockdown	21 (23.3)	8 (16.7)	13 (31.0)		
Working from the workplace	19 (21.1)	14 (29.2)	5 (11.9)		
Smart-working	31 (34.4)	17 (35.4)	14 (33.3)		
Work activity suspended: paid leave	4 (4.4)	4 (8.3)	0 (0.0)		
Work activity suspended: welfare system integration/state subsidy	13 (14.4)	5 (10.4)	8 (19.0)		
Not working due to lockdown	2 (2.2)	0 (0.0)	2 (4.8)		
**Father’s working status during lockdown (%)**				2.29 (4)	0.683
Homemaker/not employed since before lockdown	0 (0.0)	0 (0.0)	0 (0.0)		
Working from the workplace	31 (34.4)	17 (35.4)	14 (33.3)		
Smart-working	26 (28.9)	16 (33.3)	10 (23.8)		
Work activity suspended: paid leave	6 (6.7)	3 (6.3)	3 (7.1)		
Work activity suspended: welfare system integration/state subsidy	18 (20.0)	7 (14.6)	11 (26.2)		
Not working due to lockdown	9 (10.0)	5 (10.4)	4 (9.5)		
**Family member(s) infected with COVID-19 (%)**				5.30 (4)	0.258
None	79 (87.8)	40 (83.3)	39 (92.9)		
Recovered	1 (1.1)	1 (2.1)	0 (0.0)		
Taken care of at home	6 (6.7)	4 (8.3)	2 (4.8)		
Taken care of at the hospital	3 (3.3)	3 (6.3)	0 (0.0)		
Deceased	1 (1.1)	0 (0.0)	1 (2.4)		
**Friend(s) infected with COVID-19 (%)**				4.33 (4)	0.364
None	36 (40.0)	17 (35.4)	19 (45.2)		
Recovered	11 (12.2)	8 (16.7)	3 (7.1)		
Taken care of at home	10 (11.1)	4 (8.3)	6 (14.3)		
Taken care of at the hospital	13 (14.4)	9 (18.8)	4 (9.5)		
Deceased	20 (22.2)	10 (20.8)	10 (23.8)		

Bold *p*-values indicate significant differences between groups.

**Table 3:** Pearson correlation coefficients (*p*-value) regarding associations between clinical variables at T0 (language and social communication skills, emotional and behavioral profile) and CBCL 1.5–5 scales showing significantly higher T scores at T1.

	Anxious /Depressed ^a^	Aggressive Behavior ^a^	Externalizing Problems ^a^	Anxiety Problems ^a^	Oppositional Defiant Problems ^a^
**Clinical Variables Relative to Linguistic and Social Communication Skills**					
T0 Expressive vocabulary	0.187 (077)	0.084 (0.431)	0.080 (0.451)	0.135 (0.205)	0.019 (0.860)
T0 M-CHAT Failed items	0.104 (0.344)	0.097 (0.377)	0.183 (0.094)	0.105 (0.337)	0.088 (0.422)
**Pre-Emergency Psychological and Behavioral Problems**					
T0 Internalizing Problems	0.230 * (0.029)	**0.292 (0.005)**	0.168 (0.113)	**0.269 (0.010)**	**0.274 (0.009)**
T0 Externalizing Problems	0.050 (0.637)	0.069 (0.518)	−0.181 (0.088)	0.016 (0.880)	0.083 (0.437)

In bold the results that survived correction for multiple comparisons, adjusted alpha threshold *p* = 0.01. * indicated correlations significant at the uncorrected but not at the corrected alpha level. ^a^ T1-T0 difference score.

**Table 4:** Spearman correlation coefficient (*p*-value) regarding associations between environmental variables and CBCL 1.5–5 scales showing significantly higher T scores at T1.

	Anxious/Depressed ^a^	Aggressive Behavior ^a^	Externalizing Problems ^a^	Anxiety Problems ^a^	Oppositional Defiant Problems ^a^
**Socio-Demographic Information**					
SES	−0.053 (0.632)	−0.067 (0.540)	−0.147 (0.179)	0.033 (0.763)	−0.010 (0.927)
Maternal education level	−0.002 (0.983)	0.034 (0.753)	−0.131 (0.228)	0.034 (0.754)	0.065 (0.552)
Paternal education level	0.057 (0.603)	−0.086 (0.430)	−0.143 (0.187)	−0.051 (0.638)	−0.038 (0.730)
**Environmental Measures Collected during T1**					
Family member(s) infected with COVID-19	−0.064 (0.549)	−0.095 (0.375)	−0.076 (0.475)	0.086 (0.418)	−0.033 (0.757)
Friend(s) infected with COVID-19	0.121 (0.256)	0.123 (0.246)	0.084 (0.430)	0.163 (0.124)	0.115 (0.280)
Access to exteriors	−0.069 (0.521)	−0.170 (0.108)	−0.234 * (0.026)	−0.080 (0.455)	−0.237 * (0.024)
Number of rooms	−0.221 * (0.036)	−0.203 (0.054)	−0.202 (0.057)	−0.231 * (0.028)	0.177 (0.096)
Explanation(s) given to child	0.256 * (0.015)	0.122 (0.251)	0.052 (0.627)	0.033 (0.754)	0.145 (0.172)
Contact with kindergarten	−0.111 (0.305)	−0.150 (0.165)	**−0.287 (0.007)**	−0.071 (0.512)	−0.004 (0.971)

In bold the results that survived correction for multiple comparisons, adjusted alpha threshold *p* = 0.01; * indicated correlations significant at the uncorrected but not at the corrected alpha level. ^a^ T1-T0 difference score.

## Data Availability

The data that support the findings of this study are available from the corresponding author, C.C., upon reasonable request.

## Supplementary Materials

The following are available online at https://www.mdpi.com/article/10.3390/brainsci11040477/s1, Table S1: CBCL 1.5–5 T scores: Mean (Standard Deviation) and group comparisons at T0, Table S2: CBCL 1.5–5 T scores: Mean (Standard Deviation) and group comparisons at T1.
