# Supplementary material for: Effects of COVID-19 Lockdown on the Emotional and Behavioral Profiles of Preschool Italian Children with and without Familial Risk for Neurodevelopmental Disorders
Source: Brain Sci. 2021 Apr 9;11(4):477. doi: 10.3390/brainsci11040477 (PMC8070543; doi:10.3390/brainsci11040477)
Supplement: Supplementary file 1 [file brainsci-11-00477-s001.pdf]

Table S1 CBCL 1.5-5 T-scores: Mean (Standard Deviation) and group comparisons at T0

T-scores on the CBCL scales (T0)					
	**TD (*N* = 48)**	**HR-NDD (*N* = 42)**	***t* (df)**	***p***	**Cohen's *d***
**Emotionally Reactive**	52.04 (4.47)	52.21 (4.00)	-0.192 (88)	.848	-.041
**Anxious/Depressed**	51.46 (3.27)	51.19 (2.10)	0.455 (88)	.651	.096
**Somatic Complaints**	52.71 (5.49)	52.93 (4.53)	-0.206 (88)	.837	-.043
**Withdrawn**	52.21 (4.52)	52.31 (4.84)	-0.103 (88)	.919	-.022
**Sleep Problems**	53.77 (4.57)	53.19 (4.00)	0.637 (88)	.526	.135
**Attention Problems**	52.25 (3.73)	52.90 (4.68)	-0.738 (88)	.463	-.156
**Aggressive Behavior**	51.31 (3.42)	52.26 (3.90)	-1.220 (88)	.226	-.260
**Internalizing Problems**	43.31 (9.90)	45.17 (8.01)	-0.968 (88)	.336	-.205
**Externalizing Problems**	44.92 (8.33)	46.10 (9.26)	-0.636 (88)	.527	-.134
**Total Problems**	44.46 (8.75)	46.14 (8.52)	-0.922 (88)	.359	-.195
**Affective Problems**	52.52 (3.58)	53.19 (4.20)	-0.816 (88)	.417	-.172
**Anxiety Problems**	51.94 (3.50)	51.29 (2.18)	1.232 (88)	.222	.253
**Pervasive Developmental Problems**	52.83 (5.45)	53.26 (5.63)	-0.367 (88)	.715	-0.77
**Attention Deficit/ Hyperactivity Problems**	52.13 (3.13)	53.10 (5.15)	-1.062 (88)	.292	-.231
**Oppositional Defiant Problems**	51.42 (4.04)	51.24 (2.59)	0.246 (88)	.806	.052

Table S2. CBCL 1.5-5 T-scores: Mean (Standard Deviation) and group comparisons at T1

T-scores on the CBCL scales (T1)					
	**TD (*N* = 48)**	**HR-NDD (*N* = 42)**	***t* (df)**	***p***	**Cohen's *d***
**Emotionally Reactive**	54.25 (8.43)	52.21 (4.44)	1.458 (88)	.149	.297
**Anxious/Depressed**	54.21 (8.63)	51.88 (3.27)	1.732 (88)	.088	.348
**Somatic Complaints**	51.44 (3.54)	51.10 (2.59)	0.517 (88)	.606	.109
**Withdrawn**	53.58 (5.56)	52.14 (3.63)	1.473 (88)	.155	.303
**Sleep Problems**	52.98 (5.98)	53.98 (7.06)	-0.726 (88)	.470	-.153
**Attention Problems**	53.06 (4.61)	53.33 (5.53)	-0.254 (88)	.800	-.054
**Aggressive Behavior**	54.54 (8.59)	53.50 (7.47)	0.610 (88)	.544	.129
**Internalizing Problems**	43.85 (13.49)	44.36 (8.78)	-0.212 (88)	.833	-.044
**Externalizing Problems**	48.44 (12.00)	47.38 (11.44)	0.426 (88)	.671	.090
**Total Problems**	46.15 (12.35)	46.21 (9.89)	-0.029 (88)	.977	-.006
**Affective Problems**	53.00 (5.31)	52.57 (4.42)	0.413 (88)	.681	.086
**Anxiety Problems**	54.77 (9.74)	52.52 (3.96)	1.466 (88)	.148	.295
**Pervasive Developmental Problems**	52.19 (5.21)	52.26 (4.17)	-0.074 (88)	.941	-.016
**Attention Deficit/ Hyperactivity Problems**	53.48 (5.03)	53.21 (5.18)	0.245 (88)	.806	.052
**Oppositional Defiant Problems**	53.54 (5.79)	52.55 (6.23)	0.784 (88)	.435	.166
